# TFPI2 in tumor metastasis: a double-edged sword with clinical implications

**DOI:** 10.1080/15384047.2026.2678642

**Published:** 2026-05-30

**Authors:** Zhanli Guo, Yuanke Luo, Jinyu Wen, Yifang Jiang, Qixuan Kuang, Qiong Ma, Chuan Zheng, Xueke Li, Fengming You, Xi Fu

**Affiliations:** a TCM Prevention and Treatment of Metabolic and Chronic Diseases Key Laboratory of Sichuan Province, Hospital of Chengdu University of Traditional Chinese Medicine, Chengdu, China; b Oncology Teaching and Research Department, Chengdu University of Traditional Chinese Medicine, Chengdu, China; c Institute of Oncology, Chengdu University of Traditional Chinese Medicine, Chengdu, China

**Keywords:** TFPI2, tumor metastasis, bidirectional regulation, tumor microenvironment, extracellular matrix remodeling

## Abstract

Tissue factor pathway inhibitor 2 (TFPI2), a serine protease inhibitor, plays a multifaceted role in tumor metastasis. Traditionally viewed as a metastasis suppressor, it inhibits extracellular matrix (ECM) remodeling, epithelial-mesenchymal transition, and angiogenesis. However, emerging evidence indicates that TFPI2 promotes tumor progression in glioblastoma, melanoma, and other cancers by fostering an immunosuppressive tumor microenvironment, mediating pathological ECM remodeling, and enhancing angiogenesis and hematogenous dissemination. Notably, even within the same metastatic cascade, TFPI2 exhibits divergent expression patterns and context-dependent opposing functions, acting either as a metastasis suppressor or promoter. This review summarizes its context-dependent regulatory mechanisms, investigates the underlying basis from structural duality, microenvironmental heterogeneity, and receptor differences, and evaluates its potential as a therapeutic target. Future research should elucidate spatiotemporal microenvironmental dynamics to support early screening and precise intervention.

Tumor metastasis is the underlying cause of over 90% of cancer-related deaths, and dissecting its regulatory mechanisms carries substantial basic and clinical implications. Tumor metastasis encompasses a series of coordinated events, including basement membrane breach, epithelial-mesenchymal transition (EMT), angiogenesis, hematogenous dissemination, and immune evasion.^1^ ^,^ ^2^ Extracellular matrix (ECM) homeostasis plays a central role in driving tumor metastasis.^3^ Tissue factor pathway inhibitor 2 (TFPI2), a serine protease inhibitor enriched in the ECM, contains a characteristic Kunitz domain that enables it to inhibit the activities of plasmin, trypsin, and matrix metalloproteinases (MMPs). Through these inhibitory functions, TFPI2 is deeply involved in the dynamic regulation of the tumor microenvironment (TME) and is considered to be a key regulator in tumor metastasis cascade.^4^ ^,^ ^5^

Since Rao et al. first reported in 1998 that TFPI2 can significantly inhibit fibrosarcoma invasion by suppressing plasmin activity, TFPI2 has long been regarded as a classical tumor metastasis suppressor.^6^ However, with the deepening understanding of the TME and advances in high-resolution proteomics, this traditional view is increasingly being challenged. Growing evidence indicates that TFPI2 exhibits pronounced pro-metastatic properties in tumors such as glioblastoma and melanoma.^7-9^ Our previous studies have also revealed aberrantly high expression of TFPI2 in colorectal cancer tissues, with further upregulation observed in patients with metastatic disease, suggesting a driving role of TFPI2 in colorectal cancer progression.^10^ A systematic review of the literature further demonstrates that even within the metastatic process of the same tumor type, TFPI2 can display bidirectional changes in expression levels and exert dual functions—either inhibitory or promotive.^7^ ^,^ ^10-12^ This seemingly “paradoxical” functional profile underscores its complex context-dependent nature. Elucidating these intricate networks and bidirectional regulatory mechanisms is critical for advancing translational research and clinical applications.

Based on this, this article systematically reviews the differential expression of TFPI2 across various cancers and its intrinsic association with clinical prognosis. It focuses on analyzing the mechanistic roles of TFPI2 in key steps of tumor metastasis, including ECM remodeling, EMT, and angiogenesis, as well as in regulation of the tumor immune microenvironment (TIME). Furthermore, the underlying mechanisms of TFPI2's bidirectional regulatory functions are explored from perspectives such as its structural duality, TME heterogeneity, variability in tumor cell surface receptors, and current technical limitations. Finally, we address the clinical translational prospects of TFPI2 as a potential therapeutic target, alongside the key challenges and future directions in this field. This article aims to provide a comprehensive reference for elucidating the complex role of TFPI2 in tumor metastasis and a theoretical basis for the development of diagnostic and therapeutic strategies targeting TFPI2.

## Distribution and function of TFPI2

TFPI2, a highly glycosylated secreted matrix protein, is located on human chromosome 7q21.3. Its mature polypeptide chain consists of a short acidic N-terminus, three tandem Kunitz-type protease inhibitor domains (KD1-3), and a C-terminal tail rich in basic amino acids.^13^ Among these, KD1 serves as the functional core for protease inhibition, while the C-terminal tail possesses the ability to bind to cell surfaces or the ECM. Together, both domains determine the structural duality of TFPI2.^14^ Under physiological conditions, TFPI2 is expressed in multiple types of cells, ranging from placental trophoblasts, vascular endothelial cells, fibroblasts, to smooth muscle cells.^14^ Taking endothelial cells as an example, most endogenous TFPI2 is secreted extracellularly and accumulates in the local microenvironment by binding to ECM components via its C-terminal structure, thereby maintaining basement membrane homeostasis through inhibition of proteases such as MMPs.^15^ Additionally, TFPI2 can induce cellular apoptosis by upregulating caspase-3 expression and regulate the vascular endothelial growth factor (VEGF) signaling axis in a heparin-binding dependent manner, contributing to coagulation and vascular homeostasis.^16^ ^,^ ^17^ These physiological functions may be altered under pathological conditions. For instance, in atherosclerosis, TFPI2 promotes plaque stability by limiting MMP-driven matrix degradation; conversely, in pre-eclampsia, its pathological upregulation restricts trophoblast invasion, thereby contributing to disease pathogenesis.^18-20^ These studies indicate that TFPI2 exhibits functional heterogeneity in maintaining ECM homeostasis and regulating cellular invasion, a characteristic closely related to its bidirectional tumor-suppressive or tumor-promoting roles during tumor metastasis.

During tumor progression, TFPI2 plays a bidirectional regulatory role. On the one hand, TFPI2 functions as a tumor suppressor by inhibiting metastasis. Its promoter region is rich in cytosine-phosphate-guanine (CpG) islands, which in multiple malignancies such as lung cancer, cervical cancer, and bladder cancer are frequently subject to aberrant hypermethylation mediated by DNA methyltransferases (DNMTs), leading to transcriptional silencing.^21-23^ Functionally, ECM-enriched TFPI2 suppresses EMT via maintaining basement membrane homeostasis, specifically by antagonizing MMP activity, inhibiting excessive degradation of key ECM components, and reducing the proteolysis of adhesion molecules such as E-cadherin.^6^ ^,^ ^24^ ^,^ ^25^ Based on these mechanisms, TFPI2 has long been regarded as a tumor suppressor. On the other hand, recent studies have demonstrated that the expression of TFPI2 is aberrantly elevated in tumors including glioblastoma, melanoma, and colorectal cancer, where it promotes tumor metastasis by facilitating immunosuppressive tumor microenvironment (ITME) formation, pathological ECM remodeling, and hematogenous dissemination.^7^ ^,^ ^9^ ^,^ ^10^ These findings indicate that TFPI2 plays functionally heterogeneous roles in tumor metastasis.

## Mechanisms by which TFPI2 inhibits tumor metastasis

**Figure 1.**
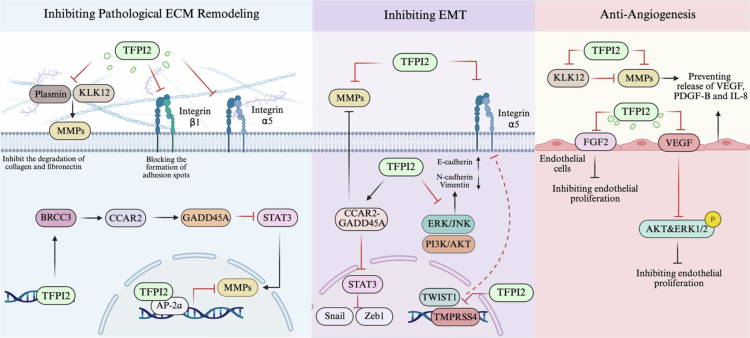
Mechanisms by which TFPI2 inhibits tumor metastasis.

The tumor metastasis cascade encompasses multiple critical steps, including ECM remodeling, EMT, and angiogenesis. These processes are regulated by a variety of molecules such as matrix-degrading proteases, integrins, cell adhesion molecules, and pro-angiogenic factors.^3^ ^,^ ^26^ ^,^ ^27^ Studies have demonstrated that TFPI2 inhibits tumor metastasis by modulating this molecular network. This section will systematically summarize the molecular mechanisms by which TFPI2 suppresses tumor metastasis, with a focus on the core steps of the metastatic cascade (Figure 1).

### Inhibition of pathological extracellular matrix remodeling

First, within the extracellular microenvironment, TFPI2 restricts MMP activation through its canonical serine protease inhibitor activity, thereby indirectly maintaining ECM homeostasis. In particular, TFPI2 attenuates the collagen-degrading activity of MMPs via interruption of the plasmin-mediated proteolytic cascade that converts pro-form of matrix metalloproteinases(pro-MMPs) into active forms, an effect that suppresses metastasis in fibrosarcoma and other malignancies.^6^ ^,^ ^28^ Also, TFPI2 functions by targeting other critical serine proteases. For instance, in non-small cell lung cancer, TFPI2 specifically antagonizes kallikrein-related peptidase 12 (KLK12), blocking KLK12-mediated activation of proMMP-1/3 and proteolytic processing of matrix signaling molecules, consequently restraining ECM remodeling and tumor metastasis. In contrast to that, TFPI2 deficiency upregulates MMP-1 expression, exacerbating matrix degradation.^29^ ^,^ ^30^ Beyond these indirect mechanisms, TFPI2 directly modulates MMP activity and expression in a highly tumor-specific manner. For example, it suppresses MMP-1 expression in bladder cancer at both transcriptional and translational levels. In glioblastoma, it inhibits MMP-1 and MMP-2 enzymatic activity without altering their expression. And in prostate cancer, TFPI2 not only reduces MMP-2 activity but also physically interacts with MMP-9 to suppress its activity, thereby exerting anti-metastatic effects.^31-33^

Second, beyond its extracellular protease-inhibitory effect, TFPI2 can act as an intracellular regulator to suppress MMP synthesis at both transcriptional and translational levels. This noncanonical pathway displays pleiotropic effects: in breast cancer, TFPI2 translocates to the nucleus and interacts with the transcription factor AP-2α, thereby preventing AP-2α from binding to the MMP-2 promoter and directly reducing MMP-2 transcription, which challenges the conventional view of TFPI2 as a secreted inhibitor only.^34^ Similarly, in hepatocellular carcinoma, TFPI2 acts as an upstream suppressor through modulation of the ubiquitination network: it associates with BRCA1-BRCA2-containing complex subunit 3 (BRCC3), a deubiquitinating enzyme, to attenuate the degradation of cell cycle and apoptosis regulator 2 (CCAR2), consequently upregulating growth arrest and DNA-damage-inducible protein 45α (GADD45A) expression. This cascade ultimately inhibits activation of signal transducer and activator of transcription 3 (STAT3), thereby blocking the transcription of matrix-degrading enzymes including MMPs and thereby establishing an ECM-protective barrier.^4^

In addition, beyond regulating the proteolytic network, TFPI2 maintains ECM homeostasis by suppressing integrin-mediated cell adhesion. This mechanism is cancer type-specific as well: in ovarian clear cell carcinoma, TFPI2 acts by inhibiting integrin β1 activation and clustering, thereby reducing focal adhesion formation and suppressing tumor cell anchorage to fibronectin (FN) and type I collagen, consequently restraining peritoneal dissemination.^35^ In breast cancer, by contrast, TFPI2 suppresses integrin α5 transcription through the downregulation of Twist family bHLH transcription factor 1 (TWIST1), attenuating tumor cell adhesion to core ECM components, blocking matrix remodeling, and inhibiting metastasis.^36^ ^,^ ^37^

In summary, TFPI2 forms a regulatory network that inhibits pathological ECM remodeling. Extracellularly, it suppresses MMP cascades and integrin-mediated adhesion; intracellularly, it represses transcription of ECM degradation-related genes via nuclear translocation or deubiquitination. This multi-layered system, linking intra- and extracellular spaces and coordinating proteolytic and adhesive signaling pathways, underscores the critical role of TFPI2 in maintaining matrix homeostasis and suppressing tumor metastasis.

### Inhibition of epithelial-mesenchymal transition

EMT represents the key process by which tumor cells acquire invasive and metastatic potential, hallmarked by the loss of cell polarity and impaired intercellular adhesion.^2^ TFPI2 interrupts EMT progression through multiple mechanisms involving the regulation of intracellular signaling pathways and suppression of cell adhesion. Regarding intracellular signal transduction, studies have demonstrated that TFPI2 significantly suppresses constitutive activation of the extracellular signal-regulated kinase (ERK), c-Jun N-terminal kinase (JNK), and phosphatidylinositol 3-kinase (PI3K)/protein kinase B (AKT) pathways in pancreatic and hepatocellular carcinoma. This consequently inhibits EMT, as evidenced by upregulation of the epithelial marker E-cadherin and downregulation of the mesenchymal markers N-cadherin and vimentin.^25^ ^,^ ^38^ ^,^ ^39^ Furthermore, TFPI2 inhibits STAT3 activation via the CCAR2-GADD45A axis, thereby suppressing the transcription of EMT-driving genes including zinc finger E-box binding homeobox 1 (ZEB1) and Snail, ultimately restraining hepatocellular carcinoma metastasis.^4^ ^,^ ^40^ ^,^ ^41^

Focusing on its role in cell adhesion process, TFPI2 attenuates tumor cell migratory capacity by disrupting integrin α5-mediated cell-matrix interactions. In breast cancer, TFPI2 inhibits integrin α5 transcription through downregulation of the transcription factor TWIST1, thereby suppresses EMT and tumor metastasis. Notably, TWIST1 overexpression reverses the anti-invasive effects of TFPI2, confirming its transcriptional activity.^36^ In non-small cell lung cancer, TFPI2 exerts anti-metastatic effects by suppressing transmembrane serine protease 4 (TMPRSS4) transcription, which upregulates microRNA-205 and consequently impairs integrin α5 expression.^42^ ^,^ ^43^ Collectively, these studies reveal that TFPI2 suppresses tumor metastasis by downregulating EMT driver gene expression and weakening cell-matrix adhesion through coordinated mechanisms involving intracellular signal transduction, transcriptional regulation, and cell adhesion.

### Anti-angiogenesis

TFPI2 exerts an inhibitory effect on tumor angiogenesis by regulating the stromal microenvironment and endothelial cell function. Within the stroma, TFPI2 impedes endothelial cell migration and curtails the paracrine secretion of proangiogenic factors by suppressing proteolytic cascades. Specifically, TFPI2 hinders the activation of pro-MMPs by inhibiting serine protease activity, such as plasmin, thereby preserving ECM integrity and limiting endothelial cell migration and sprouting. Consequently, this process reduces the release of ECM-bound VEGF and diminishes the activity of other proangiogenic factors, including fibroblast growth factor 2 (FGF2), ultimately inhibiting angiogenesis.^17^ ^,^ ^44^ For instance, in non-small cell lung cancer, TFPI2 targets KLK12 to suppress downstream matrix proteolysis and the release of VEGF and platelet-derived growth factor-B (PDGF-B).^29^ ^,^ ^45^ In fibrosarcoma, TFPI2 disrupts the tumor angiogenic microenvironment by inhibiting MMP activation and downregulating the expression of VEGF and interleukin-8 (IL-8).^28^ In both scenarios, the paracrine signaling of proangiogenic factors is attenuated, thereby suppressing tumor angiogenesis.

In the context of vascular endothelial cells, TFPI2 is involved in a negative feedback mechanism that modulates VEGF signaling. Under normal physiological conditions, VEGF stimulates the expression of TFPI2 in endothelial cells through the activation of the mitogen-activated protein kinase kinase (MEK)/ERK pathway. Subsequently, TFPI2 attenuates VEGF-induced phosphorylation of Akt and ERK1/2, thereby establishing a negative feedback loop that constrains angiogenesis.^17^ In contrast, this regulatory pathway is disrupted in malignant tumors due to the epigenetic silencing of TFPI2. Functional restoration experiments have shown that re-establishing TFPI2 expression via recombinant adeno-associated virus in glioblastoma markedly inhibits endothelial cell migration and tube formation, as well as decreases microvessel density (MVD).^46^ Comparable anti-angiogenic effects have been observed in various other tumor types, including malignant meningioma, pancreatic cancer, and esophageal cancer, where TFPI2 functions by inhibiting VEGF and reducing MVD.^24^ ^,^ ^47^ ^,^ ^48^ Additionally, research in hepatocellular carcinoma has demonstrated a significant inverse relationship between TFPI2 expression and CD34, a marker for MVD, further corroborating the role of TFPI2 in modulating angiogenesis.^25^ Collectively, TFPI2 inhibits tumor angiogenesis not only by preventing the release of ECM-bound pro-angiogenic factors during matrix degradation but also by suppressing the VEGF and FGF-2 cascades in a synergistic manner.

Throughout the metastatic cascade, TFPI2 acts beyond its capacity as a protease inhibitor, employing multidimensional mechanisms to synergistically suppress tumor metastasis. Extracellularly, it impedes pathological ECM remodeling and paracrine angiogenic signaling while disrupting cell-matrix adhesion. Intracellularly, it inhibits the transcriptional hub of EMT. This integration of extracellular and intracellular actions, encompassing proteolysis, cell adhesion, migration, and signaling pathways, underscores TFPI2's pivotal role in restraining tumor metastasis (Figure 1).

## Mechanisms by which TFPI2 promotes tumor metastasis

As discussed, TFPI2 suppresses metastasis in a wide range of cancers. Nevertheless, emerging evidence indicates that in certain tumor types or under particular microenvironmental contexts, TFPI2 conversely promotes tumor progression by facilitating the formation of an ITME and driving pathological ECM remodeling.^7^ ^,^ ^8^ ^,^ ^49^

### Promote the formation of the immunosuppressive tumor microenvironment

Existing studies have shown that TFPI2 promotes macrophage polarization toward the M2 phenotype and drives the formation of an ITME through two pathways, including paracrine signaling and intracellular transcriptional regulation. As for paracrine signaling, secreted by glioblastoma stem cells (GSCs), TFPI2 acts as a signaling ligand independent of matrix MMPs and directly binds to integrin αV (CD51) receptors on the surface of microglial cells, thereby activating the downstream STAT6 pathway and inducing M2 polarization of macrophages. The polarized M2 macrophages then suppress CD8-positive T lymphocyte (CD8+T) infiltration, promoting ITME formation.^7^ Notably, targeted blockade of TFPI2 combined with anti-programmed death-1 (PD-1) therapy significantly prolongs mouse survival.^7^ Furthermore, TFPI2 has been validated to act as a ligand in multiple cancer types. For example, in liver cancer, stroma-derived TFPI2 binds to the tissue factor–activated factor VII (TF-FVIIa) receptor complex on tumor cell surfaces, synergistically promoting tumor invasion.^8^ Mechanistically, TF-FVIIa further cleaves and activates the protease-activated receptor 2 (PAR2) signaling pathway, which induces the expression of several pro-inflammatory cytokines, including interleukin-6 (IL-6) and tumor necrosis factor-α (TNF-α). This cascade subsequently recruits and activates immunosuppressive cells, such as myeloid-derived suppressor cells (MDSCs).^50^ ^,^ ^51^ Thus, the interaction between TFPI2 and TF-FVIIa represents a key upstream signaling event driving the formation and maintenance of the ITME.

Furthermore, existing research indicates that TFPI2 can induce M2 macrophage polarization through intracellular transcriptional regulation. Specifically, under conditions of metabolic stress, TFPI2 translocates to the nucleus, where it interacts with AP-2α, alleviating its transcriptional repression of peroxisome proliferator-activated receptor γ (PPARγ), thus promoting M2 macrophage polarization.^20^ While this evidence is primarily derived from cardiovascular studies, the TFPI2-AP-2α-PPARγ pathway may also have significant implications within the TME. Hypoxia and acidosis, arising from metabolic stress, may induce M2 polarization of tumor-associated macrophages (TAMs) via this pathway. The polarized M2 macrophages subsequently secrete transforming growth factor-β (TGF-β) and VEGF, which not only drive EMT and angiogenesis but also further promote ITME formation and tumor metastasis.^52^ Co-expression analysis in cutaneous melanoma provides supporting evidence for this mechanism, revealing a strong correlation between TFPI2 and both the cytokine-cytokine receptor interaction pathway and the TNF signaling pathway, highlighting its important role in ITME remodeling.^9^

To summarize, TFPI2 drives macrophage polarization toward the M2 phenotype through dual pathways, intercellular signaling via ligand-receptor interactions and intracellular transcriptional regulation, thereby mediating immune evasion. As a key molecular link between metabolic stress and malignant tumor metastasis, TFPI2 serves as a potential therapeutic target for reversing tumor immune tolerance and inhibiting tumor metastasis.

### Promotion of pathological extracellular matrix remodeling

TFPI2 can both maintain ECM homeostasis by inhibiting protease activity and act as a signaling molecule to promote pathological ECM remodeling, thereby driving tumor metastasis. First, in the context of matrix degradation, TFPI2 promotes pathological ECM remodeling by activating MMP-2, which drives metastasis in highly aggressive melanoma.^49^ Second, in terms of transcriptional signaling regulation, TFPI2 in glioblastoma activates the JNK signaling pathway, promoting STAT3 phosphorylation, which in turn regulates EMT-related transcription factors such as ZEB1 and Snail, drives the EMT transcriptional network, maintains GSC stemness, and promotes pathological ECM remodeling.^7^ ^,^ ^53^ Meanwhile, TFPI2 can act as a paracrine signaling ligand by binding to the TF-FVIIa complex on the surface of tumor cells, synergistically promoting liver cancer invasion.^8^ TF-FVIIa serves as a key regulatory node driving tumor invasion. On one hand, it cleaves ephrin type-A receptor 2 (EphA2), activating the RAS homolog family member A (RhoA)/Rho-associated protein kinase (ROCK) pathway and inducing retraction fiber formation.^54^ On the other hand, it cleaves and activates PAR2, recruiting β-arrestin and driving cofilin dephosphorylation.^51^ ^,^ ^55^ The binding of TFPI2 to TF-FVIIa synergistically enhances these signals, thereby conferring migratory capacity to tumor cells and promoting pathological ECM remodeling.

In summary, TFPI2 promotes pathological ECM remodeling and tumor metastasis through multiple pathways, including activation of MMP-2, regulation of the STAT3-EMT signaling axis, and TF-FVIIa-mediated cytoskeletal remodeling.

### Induction of tumor angiogenesis and hematogenous dissemination

TFPI2 drives pathological vascular remodeling by maintaining the stemness of GSCs, while establishing a compensatory blood supply network through tumor cell-vascular adhesion and vasculogenic mimicry (VM), thereby promoting hematogenous dissemination of tumor cells.

In glioblastoma, TFPI2 maintains GSCs stemness by activating the JNK/STAT3 signaling pathway.^7^ GSCs exhibit high phenotypic plasticity. For instance, driven by key endothelial development transcription factors such as ETS variant 2 (ETV2), which is highly expressed in glioblastoma, GSCs can transdifferentiate into tumor-derived endothelial cells via lineage reprogramming, forming structurally abnormal but partially perfusable blood vessels.^56^ Additionally, TGF-β secreted by vascular endothelial cells induces GSCs to differentiate into pericyte-like cells, contributing to the maintenance of vascular stability and perfusion function.^57^ These findings suggest that TFPI2-mediated maintenance of GSC stemness lays the cellular foundation for the transdifferentiation of GSCs into abnormal vascular wall cells, thereby promoting pathological vascular remodeling and establishing vascular routes for tumor cell invasion along blood vessels.

Moreover, TFPI2 establishes non-canonical blood supply networks and promotes hematogenous dissemination by enhancing tumor cell-vascular adhesion and inducing VM. First, in melanoma, Mo et al. demonstrated that TFPI2 enhances the adhesion of tumor cells to vascular endothelium, and that TFPI2-highly expressing cells are enriched in perivascular regions. By reinforcing tumor cell adhesion to the vessel wall, TFPI2 promotes perivascular migration, a process termed vascular co-option, providing a microenvironment that supports hypoxia tolerance and resistance to anti-angiogenic therapy.^9^ Second, TFPI2 participates in inducing VM in tumor cells. Ruf et al. found that ECM-anchored TFPI2 acts as an extrinsic signal to induce morphological reprogramming of poorly differentiated melanoma cells, conferring phenotypic plasticity and enabling the formation of VM networks with microcirculatory perfusion function. Notably, targeted blockade of TFPI2 inhibits the formation of such networks.^49^ Collectively, TFPI2 drives hematogenous dissemination through two synergistic pathways: establishing a pathological vascular bed and providing a compensatory blood supply and escape route for tumor cells. Together, these mechanisms underscore the critical driving role of TFPI2 in tumor hematogenous dissemination.

**Figure 2.**
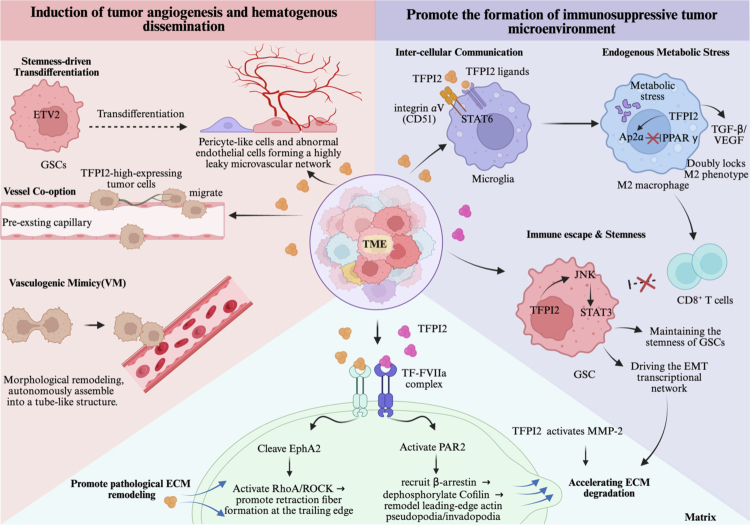
Mechanisms by which TFPI2 promotes tumor metastasis.

In terms of immune function, TFPI2 induces M2 polarization of macrophages through both paracrine and intracellular transcriptional pathways, constructing an ITME. In the stroma, TFPI2 activates MMP-2, regulates the STAT3-EMT axis, and mediates downstream TF-FVIIa signaling to promote pathological ECM remodeling. From the perspective of angiogenesis, TFPI2 maintains GSC stemness to drive vascular remodeling, enhances tumor cell-vascular adhesion, and induces VM to establish a compensatory blood supply network (Figure 2). These multidimensional mechanisms collectively constitute the regulatory network by which TFPI2 promotes tumor metastasis, positioning TFPI2 as a key regulatory node and a potential therapeutic target for anti-metastatic treatment.

## Bidirectional regulatory mechanisms and clinical applications of TFPI2

### Bidirectional regulatory mechanisms of TFPI2 in tumor metastasis

**Figure 3.**
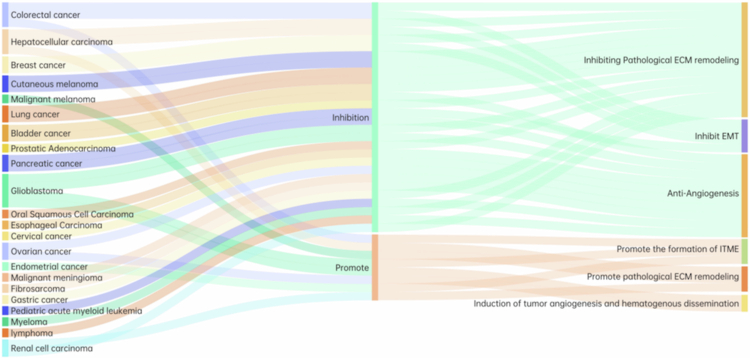
The role of TFPI2 in tumors metastasis.

Despite extensive studies on the role of TFPI2 in tumor metastasis, the intrinsic mechanisms underlying its bidirectional regulation remain incompletely elucidated. Specifically, TFPI2 acts as a metastasis suppressor in tumors such as lung cancer, breast cancer, and prostate cance,^29^ ^,^ ^30^ ^,^ ^33^ ^,^ ^34^ but becomes a metastasis promoter in endometrial cancer.^58^ ^,^ ^59^ In glioblastoma, melanoma, and colorectal cancer, TFPI2 exhibits both tumor-suppressive and tumor-promoting effects^7^ ^,^ ^9-12^ ^,^ ^60^ (Table 1, Figure 3). Notably, in ovarian cancer, there is no clear correlation between TFPI2 expression levels and its pro- or anti-tumorigenic functions.^35^ ^,^ ^61^ These findings indicate that the bidirectional regulatory characteristics of TFPI2 are not a simple consequence of expression fluctuations, but rather decided by multiple factors including its intrinsic structural duality, tumor progression stage, and the presence of receptors on tumor cell surfaces.

**Table 1.** Major findings of TFPI2 in diverse cancers.

Regulatory roles in tumor metastasis	Tumor	Tissue type	Test method	Expr.	Key findings	Ref.
Inhibit tumor metastasis	Cervical carcinoma	Cell line, tissue	qPCR, QMSP	↓	Inhibiting tumor occurrence and development after upregulation of expression	[21,62,63]
	Bladder cancer	Tissue	qPCR, IHC, Western blot	↓	The expression level is negatively correlated with tumor progression; upregulation of expression inhibits tumor cell invasion and metastasis	[23,32]
	Prostatic cancer	Cell line	RT-PCR	↓	Inhibiting tumor cell invasion and metastasis after upregulating expression	[64]
	Pancreatic ductal adenocarcinoma	Cell line, tissue	RT-PCR	↓	Inhibiting tumor proliferation, migration, and invasion after upregulation of expression	[65]
	Gastric cancer	Tissue, Serum	qMSP	↓	High methylation level and low expression correlate with poor prognosis	[66]
	Mammary cancer	Cell line, tissue	qRT-PCR, IHC	↓	Potential prognostic markers, when upregulated, inhibit tumor cell proliferation, migration, and invasion	[34,36,67]
		GOBO database	–	↑	The expression in ERα-positive tumors is significantly higher than that in negative tumors; high expression is significantly correlated with prolonged survival without distant metastasis	[68]
	Non-small cell lung cancer	Cell line, tissue	MSP, RT-PCR	↓	Low expression promotes tumor metastasis and is associated with poor prognosis	[42]
	Oral squamous cell carcinoma	Cell line, tissue	RT-PCR, MSP, Pyrosequencing, BSP, qMSP	↓	Promoting tumor occurrence, development, and metastasis through methylation	[69,70]
	Pediatric acute myeloid leukemia	Bone marrow	MSP, RRBS	↓	Associated with tumor invasive potential and malignant progression	[71]
	Myeloma	Bone marrow, cell line	MSP	↓	Hypermethylation and silencing are associated with myeloma development and progression.	[72]
	lymphoma	Tissue	qRT-PCR	↓	High-frequency methylation, diagnostic biomarker	[73]
Promote tumor metastasis	Endometrial cancer	Serum	IHC	↑	TFPI2 is associated with poor prognosis, with no significant correlation between its serum and tissue levels.	[58,59]
Bidirectional regulatory effect	Colorectal cancer	Tissue, serum	qMSP	↓	After methylation, the invasive ability of tumor cells is enhanced; diagnostic biomarkers	[11,74,75]
		Tissue	Olink, ELISA	↑	Enhancing the metastatic ability of tumor cells	[10]
	Glioblastoma	Cell line	RT-PCR	↑	Promoting glioblastoma growth; Associated with shorter patient survival; TFPI2 expression is highest in the mesenchymal subtype of glioblastoma	[7]
		Tissue	MSP	↓	After methylation, it promotes the occurrence, development, and metastasis of tumors	[12]
	Hepatocellular carcinoma	Serum, cell line, tissue	MSP, ISH, IHC	↓	Biomarkers; Low expression indicates poor prognosis; overexpression inhibits tumor occurrence and development, as well as tumor cell proliferation	[4,39,76]
		Cell line	Western blot	–	TFPI2 induces invasion through the TF-FVIIa complex signaling axis	[8]
	Invasive melanoma	Cell line	Western blot, IHC	↑	Promoting hematogenous metastasis of tumor cells	[9]
	Cutaneous melanoma	Tissue, serum	RT-PCR, MSP	↓	Promoting tumorigenesis, progression, and metastasis through methylation	[60]
	Renal cell carcinoma	Tissue	qRT-PCR, Western blot	↓	Overexpression inhibits the growth of renal cell carcinoma and induces apoptosis	[77]
		Serum	scRNA-seq	↑	Biomarkers; Differentiate patients with renal cell carcinoma; high expression is associated with poorer prognosis	[78]
	Ovarian cancer	Cell, serum	Western blot, AIA	↑	Inhibiting tumor peritoneal dissemination	[35]
		Serum	qRT-PCR, Western blot	↑	Diagnostic biomarkers; closely related to poor prognosis	[61,79]

Note: ↑ indicates upregulation, ↓ indicates downregulation.

Structurally, the bidirectional regulation of TFPI2 is tightly linked to its functional domains. The KD1 domain confers the classical serine protease inhibitor activity of TFPI2,^80^ while the C-terminal tail mediates soluble signaling ligand-like activity.^8^ In cancer tissues lacking TFPI2 receptors, soluble TFPI2 in the ECM utilizes its KD1 domain to inhibit plasmin, thereby blocking the activation of MMP cascades, maintaining basement membrane integrity, and suppressing tumor metastasi.^28^ ^,^ ^29^ Conversely, when target cells (e.g., hepatoma cells, glioblastoma-associated macrophages) express receptors such as TF-FVIIa or CD51, TFPI2 binds to the cell surface via its positively charged C-terminus and exerts signaling ligand-like activity, which activates transcription factors such as STAT6, attenuates its extracellular protease-inhibitory function, and subsequently promotes tumor metastasis and immunosuppression.^7^ ^,^ ^8^

During the early invasive stage of tumor progression, ECM degradation is necessary for tumor cells to breach the basement membrane^32^ ^,^ ^34^; however, during perivascular migration, ECM integrity becomes an important support for tumor cell survival and migration.^81^ ^,^ ^82^ This shift in ECM function provides a critical context for the bidirectional regulation of TFPI2. Specifically, during in situ invasion, tumor cells require MMP-mediated ECM degradation to penetrate the basement membrane; under these conditions, TFPI2 maintains ECM homeostasis through its protease inhibitory function, thereby suppressing metastasis.^32^ ^,^ ^34^ In contrast, during perivascular migration and amoeboid movement, tumor cell migration depends on adhesion sites and mechanical support provided by an intact ECM, and excessive ECM degradation therefore undermines this physical basis.^81^ ^,^ ^82^ In this process, TFPI2-mediated ECM homeostasis provides structural support for tumor cell adhesion and migration, creating a local microenvironment favorable for tumor cell survival and thereby enhancing tumor cell migratory capacity and anoikis resistance.

Besides, the functional plasticity of TFPI2 is influenced by post-transcriptional and post-translational modifications. For one thing, tumor cells can produce an alternatively spliced variant, asTFPI-2, which lacks a complete open reading frame or a poly(A) tail, making it difficult to form stable, translatable mature mRNA. Since asTFPI-2 shares partial exon sequences with functional TFPI2, conventional qRT-PCR assays cannot distinguish between the two without transcript-specific primer design, providing important clues for the inconsistent findings regarding TFPI2 expression levels and functions across different studies.^83^ For another, microenvironmental stress drives proteolytic cleavage of TFPI2. In hostile microenvironments such as hypoxia and acidosis, inflammatory proteases like neutrophil elastase are activated, leading to TFPI2 cleavage and generating C-terminally truncated N-terminal fragments and free peptide fragments.^84^ These fragments, lacking the C-terminal strong positive charge anchor domain, are more likely to detach from electrostatic binding to the ECM and may exhibit higher tissue diffusion capacity, potentially participating in ligand-like signaling through exposed functional sites to mediate pro-metastatic effects. Of particular interest is the cationic peptide fragment EDC34 released upon C-terminal cleavage.^84^ Given the dual-edged effects of cationic defense peptides in tumor immunity,^85^ it is plausible that within the inflammatory tumor microenvironment, EDC34 may function as a potential danger-associated molecular pattern (DAMP), thereby modulating immune responses by recruiting MDSCs or driving M2 polarization of TAMs, and ultimately promoting tumor progression through immune-related mechanisms.

### Clinical applications of TFPI2 in tumor

As a key regulator in tumor progression, TFPI2 holds considerable translational potential for the early screening and diagnosis of cancer. A study involving 351 patients demonstrated that serum TFPI2 has considerably high diagnostic efficacy for stage II–IV ovarian clear cell carcinoma (AUC = 0.815), with sensitivity and specificity superior to the conventional marker CA125 (AUC = 0.505),^86^ and the corresponding assay has been translated into clinical practice. In colorectal cancer screening, combined detection of fecal TFPI2 and syndecan 2 (SDC2) methylation significantly improves diagnostic accuracy (AUC = 0.96) and reduces the missed diagnosis rate for left-sided colon cancer and colon adenoma.^87^ Further clinical validation has also confirmed the robustness and reproducibility of this combined testing strategy across different cohorts, supporting its utility as a reliable non-invasive screening tool.^88^

Multiple studies suggest that TFPI2 has prognostic value in various malignancies. In glioblastoma and ovarian cancer, high TFPI2 expression is associated with shorter overall survival (OS) and progression-free survival, indicating poor prognosis.^7^ ^,^ ^61^ Conversely, in breast cancer and non-small cell lung cancer, low TFPI2 expression correlates with advanced tumor stage and lymph node metastasis, also suggesting poor prognosis.^42^ ^,^ ^67^ ^,^ ^89^ Additionally, in uveal melanoma, high TFPI2 expression is associated with tumor metastasis and reduced OS, whereas in cutaneous melanoma, it correlates with better survival outcomes.^9^ These differences indicate that the prognostic direction of TFPI2 is highly tumor type-dependent, and its clinical interpretation requires comprehensive evaluation based on the specific pathological context.

Hypermethylation of the TFPI2 promoter reduces its expression, representing a key mechanism underlying epigenetic silencing and providing a critical entry point for targeted therapy. In lung cancer and pancreatic cancer, demethylating agents such as 5-aza-2′-deoxycytidine and the natural active compound curcumin can reverse the hypermethylation status of the TFPI2 promoter by inhibiting DNMT activity, thereby restoring TFPI2 expression and exerting anti-tumor effects.^38^ ^,^ ^90^ In colorectal cancer, elevated expression of ten-eleven translocation methylcytosine dioxygenase 1 (TET1) or enhanced binding to the TFPI2 promoter similarly reverses TFPI2 methylation and suppresses tumor progression.^91^ Of note, given the bidirectional regulatory characteristics of TFPI2 in tumors, its targeting strategy requires comprehensive assessment of tumor type and microenvironmental features to avoid potential pro-tumorigenic effects and improve therapeutic safety.

## Summary and outlook

This article systematically reviews the mechanisms by which TFPI2 suppresses and promotes tumor metastasis, demonstrating that its functional orientation is shaped by its structural duality and TME heterogeneity, rather than being a simple consequence of its expression level. The N-terminal Kunitz domain mediates protease inhibitory function, whereas the C-terminal cationic tail confers signaling ligand-like activity. Meanwhile, additional factors, such as differential receptor expression of tumor cells, tumor progression stage, proteolytic processing and post-translational modifications, further clarify the functional consequence of TFPI2.Despite extensive research, the key mechanisms underlying the bidirectional regulation of TFPI2 remain poorly understood. Several significant gaps persist in our understanding: the in vivo activation and biological functions of its various domains remain ambiguous; there is an absence of direct evidence supporting the role of aberrant splice variants and proteolytic fragments in functional transitions; and the receptor-dependent signaling networks within distinct tumor microenvironments have not been systematically characterized. Future research should adopt a comprehensive approach that integrates structural analysis, microenvironmental context, and functional outcomes. For foundational studies, methodologies such as spatial transcriptomics, single-cell sequencing, high-resolution proteomics, and patient-derived organoids should be utilized to meticulously analyze the spatiotemporal distribution and functional states of full-length TFPI2, along with its aberrant splice variants and degradation fragments throughout tumor progression. Meanwhile, it is imperative to investigate domain-specific functions, with a particular emphasis on the C-terminal mediated receptor binding interface and its associated downstream signaling pathways, to elucidate the molecular mechanisms underlying TFPI2-driven pro-metastatic effects. From a clinical translation perspective, developing targeted intervention strategies that selectively inhibit the C-terminal pro-tumor signaling while preserving the N-terminal protease inhibitory activity presents significant potential for anti-metastatic therapy. Considering the pivotal role of TFPI2 in modulating the TIME, integrating TFPI2-targeted regulation with immune checkpoint inhibitors may provide a novel strategy to counteract immune evasion. Overall, elucidating the mechanisms of TFPI2 within different tumor niches will facilitate its precise application as both a diagnostic biomarker and a therapeutic target.

## Data Availability

No datasets were generated or analyzed during the current study.

## Abbreviations

AKT: protein kinase B
asTFPI-2: alternatively spliced TFPI-2
BRCC3: BRCA1-BRCA2-containing complex subunit 3
CCAR2: cell cycle and apoptosis regulator 2
CD51: integrin alpha V (CD51)
CD8+ T: CD8-positive T lymphocyte
CpG: cytosine-phosphate-guanine
DAMP: danger-associated molecular pattern
DNMTs: DNA methyltransferases
ECM: extracellular matrix
EMT: epithelial-mesenchymal transition
EphA2: ephrin type-A receptor 2
ERK: extracellular signal-regulated kinase
ETV2: ETS variant 2
FGF2: fibroblast growth factor 2
FN: fibronectin
GADD45A: growth arrest and DNA-damage-inducible protein 45α
GSCs: glioblastoma stem cells
IL-6: interleukin-6
IL-8: interleukin-8
ITME: immunosuppressive tumor microenvironment
JNK: c-Jun n-terminal kinase
KD1-3: Kunitz-type protease inhibitor domains 1–3
KLK12: kallikrein-related peptidase 12
MDSCs: myeloid-derived suppressor cells
MEK: mitogen-activated protein kinase kinase
MMPs: matrix metalloproteinases
MVD: microvessel density
OS: overall survival
PAR2: protease-activated receptor 2
PD-1: programmed death-1
PDGF-B: platelet-derived growth factor-B
PI3K: phosphatidylinositol 3-kinase
PPARγ: peroxisome proliferator-activated receptor gamma
pro-MMPs: pro-form of matrix metalloproteinases
RhoA: RAS homolog family member A
ROCK: Rho-associated protein kinase
SDC2: syndecan 2
STAT3: signal transducer and activator of transcription 3
TAMs: tumor-associated macrophages
TET1: ten-eleven translocation methylcytosine dioxygenase
TF-FVIIa: tissue factor-activated factor VII
TFPI2: tissue factor pathway inhibitor 2
TGF-β: transforming growth factor-beta
TIME: tumor immune microenvironment
TME: tumor microenvironment
TMPRSS4: transmembrane serine protease 4
TNF-α: tumor necrosis factor-alpha
TWIST1: twist family bHLH transcription factor 1
VEGF: vascular endothelial growth factor
VM: vasculogenic mimicry
ZEB1: zinc finger E-box binding homeobox 1
